# A Wireless EEG Recording Method for Rat Use inside the Water Maze

**DOI:** 10.1371/journal.pone.0147730

**Published:** 2016-02-01

**Authors:** Richard C. Pinnell, Rand K. Almajidy, Robert D. Kirch, Jean C. Cassel, Ulrich G. Hofmann

**Affiliations:** 1 Neuroelectronic Systems, Dept. of Neurosurgery, University Medical Centre Freiburg, Freiburg, Germany; 2 Institute for Signal Processing, University of Luebeck, Luebeck, Germany; 3 College of Medicine, University of Diyala, Diyala, Iraq; 4 Université de Strasbourg, Laboratoire de Neurosciences Cognitives et Adaptatives (LNCA), F-67000, Strasbourg, France; 5 CNRS, LNCA UMR 7364, F-67000, Strasbourg, France; Tokai University, JAPAN

## Abstract

With the continued miniaturisation of portable embedded systems, wireless EEG recording techniques are becoming increasingly prevalent in animal behavioural research. However, in spite of their versatility and portability, they have seldom been used inside water-maze tasks designed for rats. As such, a novel 3D printed implant and waterproof connector is presented, which can facilitate wireless water-maze EEG recordings in freely-moving rats, using a commercial wireless recording system (W32; Multichannel Systems). As well as waterproofing the wireless system, battery, and electrode connector, the implant serves to reduce movement-related artefacts by redistributing movement-related forces away from the electrode connector. This implant/connector was able to successfully record high-quality LFP in the hippocampo-striatal brain regions of rats as they undertook a procedural-learning variant of the double-H water-maze task. Notably, there were no significant performance deficits through its use when compared with a control group across a number of metrics including number of errors and speed of task completion. Taken together, this method can expand the range of measurements that are currently possible in this diverse area of behavioural neuroscience, whilst paving the way for integration with more complex behaviours.

## Introduction

The ability to wirelessly record EEG in freely-moving animals has resulted in numerous improvements for behavioural testing, which have otherwise relied on traditional tethered recording techniques. In addition to their rapid set-up times, wireless systems can be utilised and moved between numerous different arena types, without having to consider custom-designed tethered recording solutions for each different arena configuration; that typically involve swivels, counterweights, rods and commutators. Crucially, they are able to circumvent various practical issues inherent with tethered recording, which includes cable twisting/snagging, the external force and visual distraction arising from the cable itself, as well as 50 Hz noise pickup. In addition, wireless systems are ideal in situations where a cable tether would be deemed impractical, e.g. 3D maze, multiple arenas, or arenas with enclosed sections. Despite their versatility, there have to date been no documented successful attempts of carrying out wireless multichannel recording inside a water maze. Being able to successfully do so would expand the range of measurements that are possible in this large and diverse area of behavioural neuroscience.

The water maze environment presents numerous complications to EEG recording; primarily of which involves a) the tenacity of water to get everywhere, and b) the excessive shaking behaviour of rats inside the maze. Recordings of neuronal activity inside a water maze have nonetheless been previously attempted using tethered recording systems. Hollup et al. [1,2], and later Fyhn et al. [3] were able to carry out such recordings inside an annular (ring-shape) water-maze task using Vaseline to coat the tether/headstage interface and protect it from the surrounding water medium. However this method has been later criticized to be inadequate by Korshunov and Averkin [4], given the tendency for rats to dive underwater. These researchers designed the first purpose-built microdrive for waterproof tethered recordings inside a Morris Water Maze [4], which was later followed by a multichannel version with in-built preamplifiers [5]. Whilst this device was shown to perform adequately inside a circular arena type, there exists the possibility of practical issues when attempting such tethered system solutions for larger and more complex maze types, e.g. the double-H maze (e.g. [6,7]), which contains multiple arms and enclosed sections.

There are numerous implantable EEG recording system prototypes [8–10] that are inherently waterproof. Because battery changes are impractical or impossible with such systems, these systems are typically designed to operate at a low-power, in order to accommodate smaller-sized batteries at a reduced capacity. This presents a problem with the wireless transmission range, since wireless transceivers typically draw many times the current as the microcontrollers or ASIC's that control them, during a transmit cycle. For instance, the device by Chang et al. [8] can only operate inside a Faraday cage, and the device by Liu et al. [9] has a maximum specified range of just under 1 m. Furthermore, implantable devices have to transmit their signals through tissue, which further limits their practical RF range. Depending on the orientation of the system in water, the signal may pass through water, which is an effective absorber of RF signals. As such, the only implantable system documented to successfully perform wireless recording inside a water-maze was a 1-channel system with a transmission range (in water) of 30 cm [10]. This distance is unsuitable for the majority of water-maze tasks in rats, as they typically require transmission ranges of up to several meters (e.g. Morris water maze, double-H maze). As such for practical multichannel EEG recording inside such environments, novel techniques are thus required.

Another issue that is exclusive to water-maze recording are movement artefacts which arise from the rat shaking. Although this has not been documented in previous studies, the force from animal shaking inside the water maze is markedly greater than that observed inside dry mazes (e.g. from grooming), and thus required special attention when developing the method as described here.

This study demonstrates the first successful attempt at recording multichannel EEG activity inside a water-maze, using a wireless system. Female Sprague Dawley rats (n = 8) were implanted with recording electrodes in the hippocampus and striatum, along with a 3D-printed headstage socket. The socket acted as an attachment site for a modified stainless-steel thimble, which housed a commercial wireless system (W32; Multichannel Systems), along with a waterproof coating. Rats were trained in a procedural-learning version of the double-H water maze task, and their EEG was recorded before, during and after the swimming task. The wireless recording system was found not to impair the mobility of the rats, as seen when comparing the trial latencies with that of a control group (without the wireless system or socket; n = 6). Marked differences in the LFP could be seen inside the water-maze, which are consistent with well-known correlates of exploratory behaviour, including increases in theta-frequency power spectral density (PSD) [11,12] and coherence [13,14], as well as increases in dCA1 theta-gamma cross-frequency modulation (e.g. [15,16]).

## Materials and Methods

### Headstage Socket and Protective Thimble

**Fig 1:**
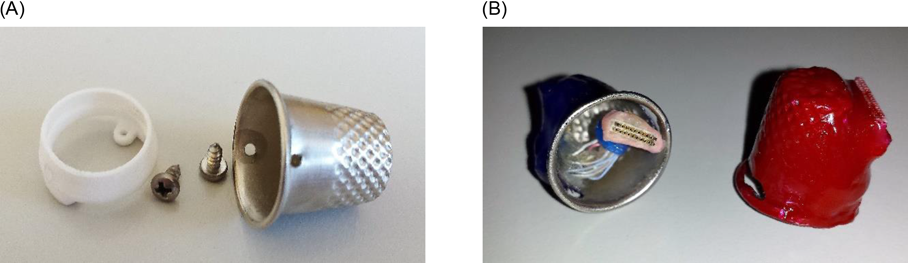
Photographs of headstage socket, and waterproof thimble attachment. A polyamide head-stage socket is shown with its connecting stainless steel thimble and screws, and also a PCB connector which is placed inside during surgery (A). A wireless system attachment was created using electrode connectors, glue, and a sewing thimble (B).

A headstage socket was designed for implantation in rats, for the purposes of housing the PCB electrode connector, and for securely mounting the waterproof wireless system attachment during behavioural experiments. This connector was previously described for facilitating the attachment of a stainless-steel protective thimble, for the group-housing of post-surgery rodents [17]. The headstage socket was designed using Solid Edge ST6 (Siemens PLM Software) and manufactured using polyamide with selective laser sintering (Beta Layout, Ireland). The socket is a 5 mm high cylinder with an internal/external diameter of 13.5/15.6 mm, respectively (Fig 1A). Two internal feet protrude 2 mm at the base, which allows for attachment to the skull using two stainless-steel mounting screws (0–80 x 1/8; Plastics One; USA). Two 1.2 mm holes were drilled at opposite ends of the headstage socket in order to facilitate attachment of the waterproof wireless system attachment using M1.4x4mm self-tapping electronic screws (Phillips). During normal housing conditions, this waterproof attachment was replaced with a standard 18×9 mm stainless-steel sewing thimble (CKB Ltd; China), which contained 1.2 mm holes drilled at either end for attachment to the headstage socket. A PCB connector was manufactured (PCB-Pool, Ireland) to fit inside the socket, and contains an 18-pin zif connector, two DIP connectors, and an 18-pin electrode connector (A79040-001; Omnetics Connector Corporation) for providing an interface between the implanted electrodes and the wireless recording system.

### Wireless System Attachment

An 18×9 mm stainless-steel sewing thimble (CKB Ltd; China) was cut in order to provide an opening at the top and side. A custom-made 3 cm connector was fabricated and contained a 36-pin male connector at one end (A79028-001; Omnetics Connector Corporation), and an 18-pin female connector at the other (A79041-001; Omnetics Connector Corporation). This connector was affixed to the thimble through this drilled opening using a clear 2-part epoxy resin (Araldite; Farnell), such that the W32 wireless system (Multichannel Systems; Reutlingen, Germany) could be plugged into the top of the thimble, and the 18-pin connector could protrude from the base (Fig 1B). Two 7 mm lines were drilled into either end of the thimble at the base using a 1.2 mm drill piece, for allowing the thimble to attach to the headstage socket using M1.4x4mm self-tapping electronic screws (Phillips). A miniature elastic band (~8 mm diameter) was added around the connecting cable to provide damping during animal movement. Furthermore, the 18-pin Omnetics connector was covered around its housing with a 2-part dental cement (Palapress; Heraeus Holding GmbH; Germany), to provide a strong grip for accessing the connector during use.

### Handling and Habituation

Prior to surgery, all rats underwent 5 days of handling in order to familiarise them with the experimenter. On days 3–5 rats were placed into the (dry) double-H maze in pairs for 10-minutes/day, in order to familiarise them with the maze environment.

### Surgery

**Fig 2:**
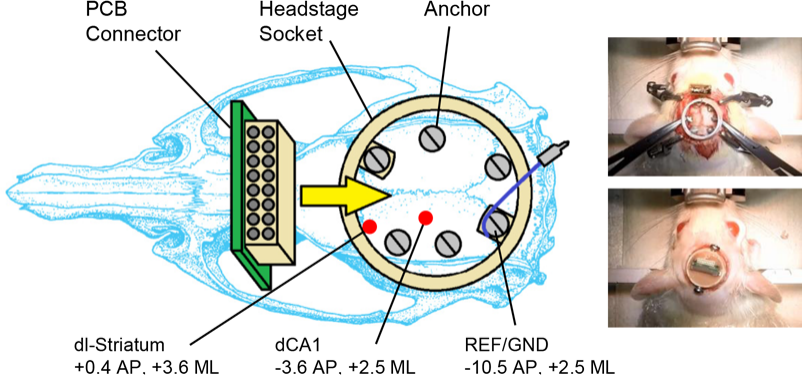
Rat skull-implant diagram for the water-maze recording group. Diagram showing the location of the headstage socket on the rat’s skull, along with the electrode and anchor positions. Photographs taken during the surgery procedure depict the position of electrodes, socket and anchors (top), and the position of the PCB connector (bottom). The control group received the electrodes, anchors and PCB connector, but no headstage socket.

The procedure for implantation followed a similar method used for implantation of the headstage socket [17]. All procedures involving animals and their care were conducted in conformity with institutional guidelines, which are in compliance with the guidelines of the German Council on Animal Protection (25.5.198) and international (NIH publication no 85–23, revised 1985) laws and policies. All protocols were approved by the Animal Care Committee of the University of Freiburg (permit 35–9185.81/G-13/97). Female Sprague-Dawley rats (300-320g; n = 14) were anaesthetised with oxygen (0.15 l.min^-1^) and isoflurane (Abbvie, USA); the latter of which was initially set to 4% and gradually lowered to 1.5% after placing the animal into the stereotaxic frame (David Kopf, USA). Animal breathing, reflexes and level of anaesthesia were monitored throughout the duration of the surgery. Animals were implanted with a Teflon-coated platinum iridium wire (140 μm diameter) in the dCA1 region of the hippocampus (AP: -3.6, ML: +2.5; from Bregma, DV: -2.2 from dura matter), and a 16-contact flexible microelectrode (Imtek; Freiburg University) in the left dorsolateral striatum (AP: +0.4, ML: +3.6; from Bregma, DV: -3.7 from dura matter). Prior to implantation, both electrodes were affixed to a 125 μm glass rod using superglue, which provided rigidity and allowed for accurate implantation [18]. In the water-maze recording group (n = 8), two stainless-steel mounting screws (0–80 x 1/8; Plastics One) were used to attach the front and rear feet of the headstage to the anterior and posterior portions of the skull, respectively (Fig 2). The posterior attachment screw (AP: -10.5, ML: +2.5; from Bregma) was pre-soldered to a connecting pin via silver wire (Science Products Gmbh, Germany), and formed the reference/ground electrode connector. Four additional mounting screws were affixed along the edges of the socket on each skull plate to provide further attachment of the implant. The recording and ground electrodes were then affixed to their respective connectors on the PCB, which was inserted into the socket with the 18-pin Omnetics connector facing upwards. A 2-compound dental cement (Palapress; Heraeus Holding GmbH; Germany) was then prepared and used to fill the headstage socket, from its base up to the top of the PCB connector. Two attachment screws were inserted at the front and rear sections of the headstage during this process, and were gradually turned as the cement had hardened, in order to prevent them from sticking to the cement. A headstage socket was not utilised in the control group (n = 6), although these animals had the same electrodes/anchors and PCB connector, which were otherwise mounted in the same position. In both cases care was taken to provide a smooth cement surface along the base of the implant on all sides; such that the skin could heal around the implant. Following surgery animals were given an injection of saline (1 ml i.p.) and placed inside a heated cage to recover. Video footage demonstrating the implantation procedure can be found at http://figshare.com/s/a06fdd80fa2411e4b38b06ec4bbcf141.

### Recovery and Euthanasia

Following surgery, rats were housed individually for 6 days, with the protective stainless-steel thimble disconnected, in order to prevent it from sticking to the blood clot. After 6 days, the protective thimble was attached to the headstage socket, and animals were reunited with their cage-mates. For the first 4 days following surgery, rats were given daily injections of the non-steroidal anti-inflammatory drug Caprofen (Carprieve; Norbrook, UK; 4 mg.kg^-1^ s.c.). Rats were weighed daily during the first 2 weeks, and every 2 days thereafter. No signs of nibbling or damage inflicted to the co-housed animal were observed to the protective thimbles or the skin around it. Instead, the group-housed animals where easier to handle and showed less signs of stress during experiments.

### Attachment of System and Waterproof Connector

**Fig 3:**
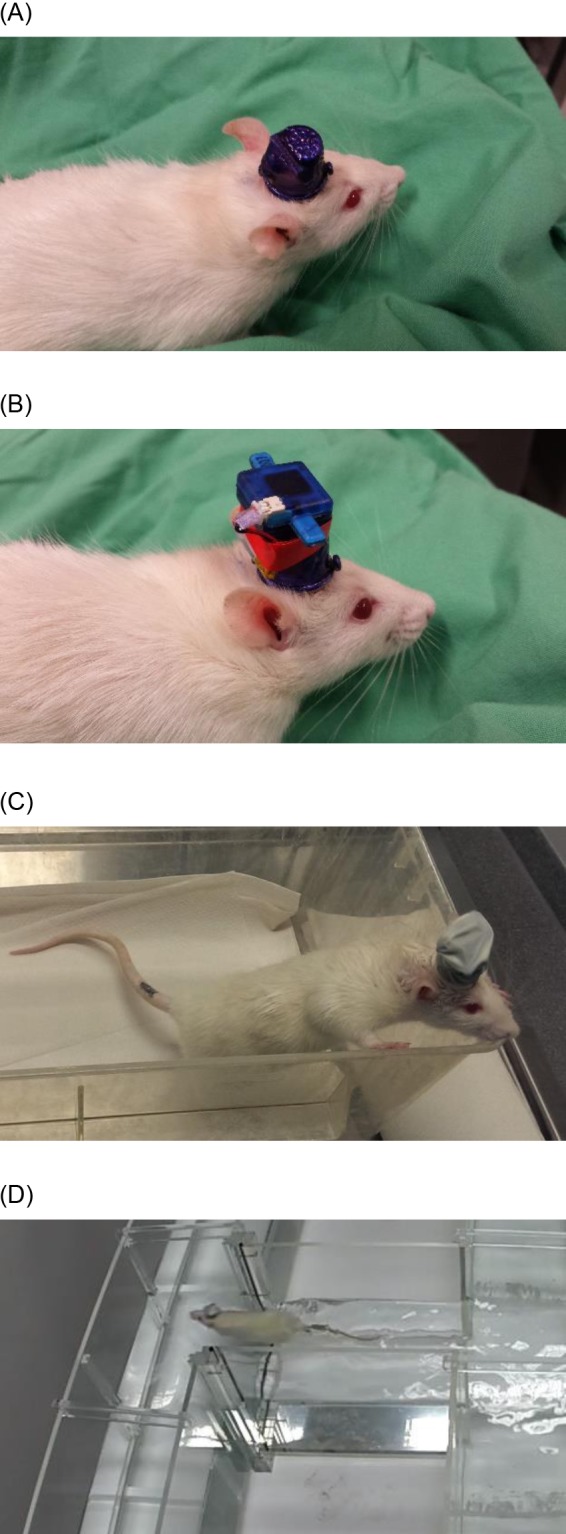
Photographs of Waterproof Connector. The wireless system connector provides stability for the wireless system, and is shown both without (A) and with (B) the W32 wireless system (Multichannel Systems Gmbh; Germany). With the waterproofing latex finger-cot (C), the rat is able to swim freely about the water maze (D) whilst LFP is transmitted wirelessly.

During each test session, the protective stainless-steel thimble was detached from rats, and reattached prior to returning them to their cage. The wireless waterproof connector was prepared by first attaching a wireless recording system (W32; Multichannel Systems) to the top of the waterproof connector. A miniature lithium-polymer battery (35 mAh; digitalo.de GmbH, Germany) was connected to the system and affixed to the side of the waterproof connector using tape (Fig 3A and 3B). A latex finger-cot (Med-Comfort) was folded over the system/connector, and held in place using a miniature elastic band. The system was then attached to the animal by a) connecting the recording cable, and b) affixing the attachment over the headstage socket and securing with screws. Vaseline was applied around the base of the thimble, and the latex finger cot was rolled down over the thimble base (Fig 3C). The system was wirelessly powered down in-between recordings, and remotely activated prior to use, in order to preserve its battery life, which was 30 minutes of continuous stable recording and transmission at 10 KHz sample rate. The total weight of the wireless system, battery, waterproof connector and waterproof coating is approximately 8 g. Following the 4 trials in a single session, the wireless system and connector were detached and the rat was dried and placed under an infra-red heat lamp, prior to returning to their cage.

### Double-H Water Maze

This experiment utilised a procedural-learning variant of the double-H water maze task (see [19]). All rats underwent an initial day of habituation (6 trials), followed by 4 days of training (4 trials / day). During habituation, the water maze was filled to a height of 17 cm, and a 10 cm diameter platform was immersed at the extremity of the NE arm such that it protruded 1 cm above the surface of the water. For each trial, rats were released from the extremity of the S arm, and were given 60 s to swim to the escape platform. Rats that did not find the platform during this time were returned to the starting position, and gently guided to the platform by the experimenter. Upon reaching the platform, rats were given 15 s to consolidate their surroundings, followed by a further 10 s inter-trial delay. During the training sessions, the platform was relocated to the NW arm, and a transparent guillotine door was placed at the entry of the N arm in order to force a right/left turn for rats. Also, the water was rendered opaque by mixing in 250 g skimmed milk powder, and raised to 19 cm such that the platform was hidden 1 cm beneath the water surface. The time taken for rats to reach both the goal arm and platform were noted, alongside the number of initial and repetitive errors. An initial error was counted whenever the rat deviated from the correct swim path into one of the 5 error zones for the first time. A repetitive error was counted if this occurred more than once for each zone.

The water maze was surrounded on all sides by high-contrast cues, and a VGA camera (Kinect; Microsoft) was mounted onto the ceiling above the maze for post-task scoring of animal behaviour. Rats in the EEG recording group were given the wireless system plus waterproof attachment before the training and habituation sessions, which was used to record and transmit EEG during two phases of the task (inside the water-maze, on the platform). Additionally, rats underwent 1 minute of pre-task baseline recording, prior to beginning their first trial. For the purpose of video-EEG synchronisation, both recording types were started simultaneously at the beginning of each and every trial inside the maze. The receiver for the wireless system was placed on a shelf which was adjacent to the north side of the maze.

### Signal Analysis

Behavioural results, including trial latencies, start/end times and error counting were performed manually using the video data from each rat. EEG data was extracted into Matlab (Mathworks Inc) using NeuroExplorer 4.0 (REF) and was organised into pre-task, maze and platform epochs using custom-written scripts and the timing information from the video recordings. Data arising from the maze was extracted as the 4 seconds prior to the rat reaching the platform. Artefacts were manually removed by combing through the all of the EEG recordings using a 0.25 s sliding window. Artefacts in this case were directly a result of the rat shaking and/or hitting the system on the maze wall, and manifested as a characteristic burst oscillation on the raw EEG trace. The extent of artefact removal was in this case characterised by comparing the lengths of the signals both with and without artefacts. Both the power-spectral density and coherence were computed using custom scripts that were adapted from the Chronux library (http://www.chronux.org); each using 3 orthogonal data tapers, and a 2 s sliding window with 1 s overlap. Power spectral density for each epoch was normalised by dividing by its root-mean-square (RMS) value, and the peak theta-frequency PSD/coherence between 5–12 Hz was extracted for further comparison.

Theta-gamma cross-frequency modulation was calculated based on a normalised entropy method [16,20] using custom-written Matlab scripts. Signals were decomposed into composite theta phase / gamma envelope signals, by first applying zero-phase delay filters, and then the Hilbert transform; which resulted in a matrix of signals covering each theta and gamma frequency band. The theta phase range was split into 18 × 20° bins, and the instantaneous gamma amplitude at each point in the signal was added to its corresponding theta-phase bin. The resulting histogram of gamma/phase values was normalised, and the signal entropy was then derived for each theta phase bin. The modulation index of each theta/gamma filtered signal pair was then obtained by normalising the entropy result with the maximum possible entropy value; which was taken as log (18).

### Statistics

Analysis of rat behaviour, including initial/repetitive errors and the time taken to reach the goal arm / platform, were analysed using a 2-way ANOVA, looking for significant differences between the test session (1, 2, 3, 4) and rat group (with/without wireless system). Comparisons of theta-frequency PSD and coherence were made using a 1-way ANOVA, which looked for significant differences between the recording arenas (pre-maze, maze, platform). The percentages of the recorded signals after artefact removal were compared using a 1-way ANOVA for each recording electrode, looking for differences between the individual recording sessions. Where significant, each analysis was followed using a Bonferroni multiple comparison test. Each matrix of modulation-index values was compared to a surrogate data distribution (100 surrogates), obtained by taking randomised trial data from randomised rats, and shifting the amplitude of the signals in time to create a composite version of each input signal. The results are displayed as a matrix of *z-*scores highlighting the significance of each MI estimate.

## Results

### Rat Behaviour

The waterproof recording attachment was able to successfully facilitate wireless, waterproof recording inside the water-maze during both the swimming and platform phases of the task (see S1–S3 Videos). Rats were able to swim freely, without any leakages or practical issues arising from the use of the waterproofed system.

**Fig 4:**
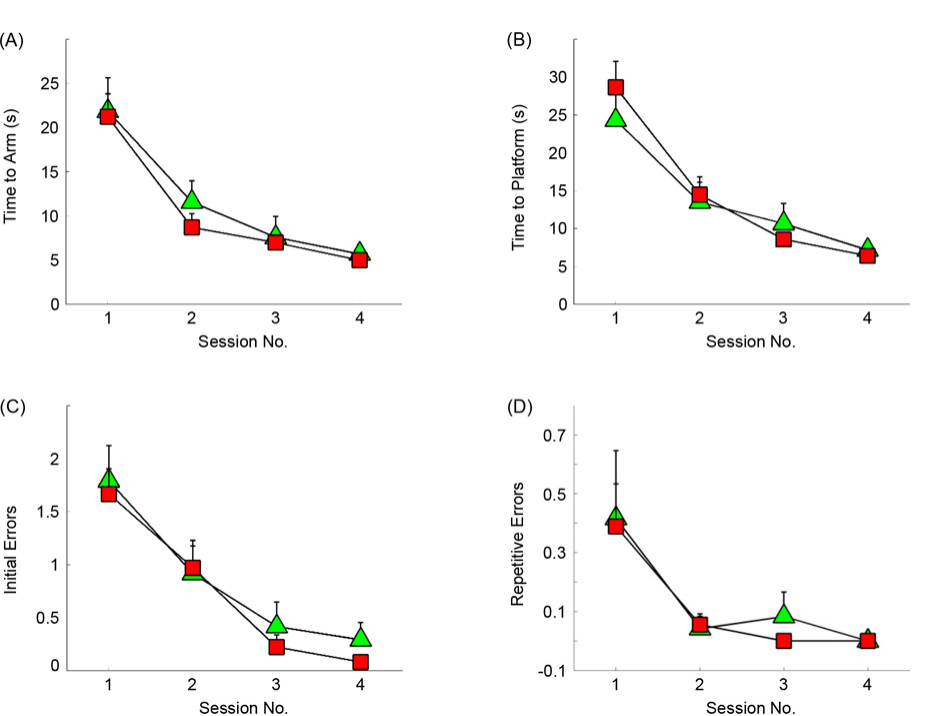
Rat behavioural performance during the water-maze task. Performance differences are shown between the wireless system (square) and non-wireless system (triangle) groups, + SEM. Both groups had shown comparable performance on all measures, including the time taken to reach the goal arm (A) and platform (B), as well as the number of initial (C) and repetitive (D) errors.

Behavioural performance for both the EEG-recording and sham groups are shown (Fig 4A–4D). Across the training sessions, a clear pattern of learning was noted in both groups of rats, as highlighted by reductions in the number of individual and repetitive errors, as well as reductions in the time taken to reach the goal-arm and platform. A 2-way ANOVA on these performances indicated a significant effect of training session on initial (F(3,52) = 21.87, p<0.0001) and repetitive errors (F(3,52) = 7.63, p<0.001), and also a significant effect of session on both goal-arm (F(3,52) = 25.68, p<0.0001) and platform latencies (F(3,52) = 26.7, p<0.0001). No significant effect of test group was found on any of these measures, highlighting a lack of changes in any of these performance measures brought about by the use of the wireless system.

### LFP Performance

**Fig 5:**
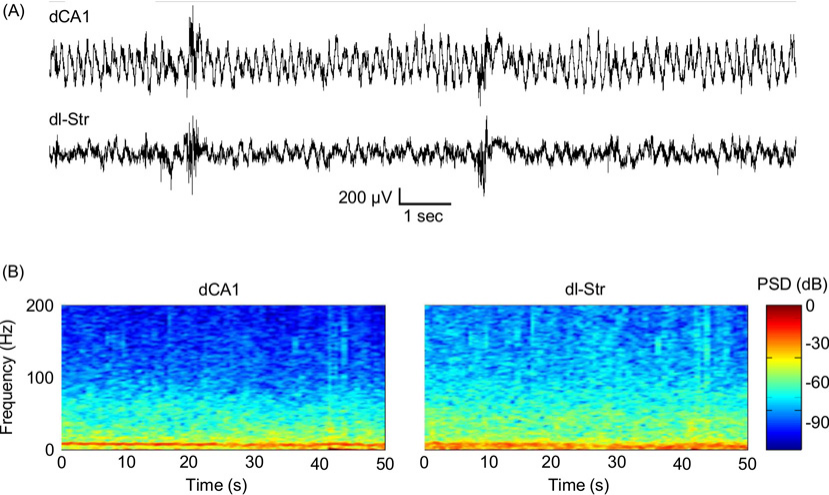
LFP recording quality inside the water maze. A representative trace from the dCA1 and striatal electrodes is shown (A), alongside representative spectrograms pertaining to a single trial inside the water maze (B). Recording quality was maintained throughout the training sessions, and the system was able to quickly recover from movement artefacts, which arose whenever the rats shook their head or hit the system on the maze wall.

A representative trace from the dCA1 and striatal electrodes from water-maze recording is shown (Fig 5A), alongside representative PSD spectrograms from a single trial (Fig 5B). Recording quality was maintained throughout the training sessions, and the system was able to quickly recover from movement artefacts, which arose whenever the rats shook their head or hit the system on the side of the maze.

**Fig 6:**
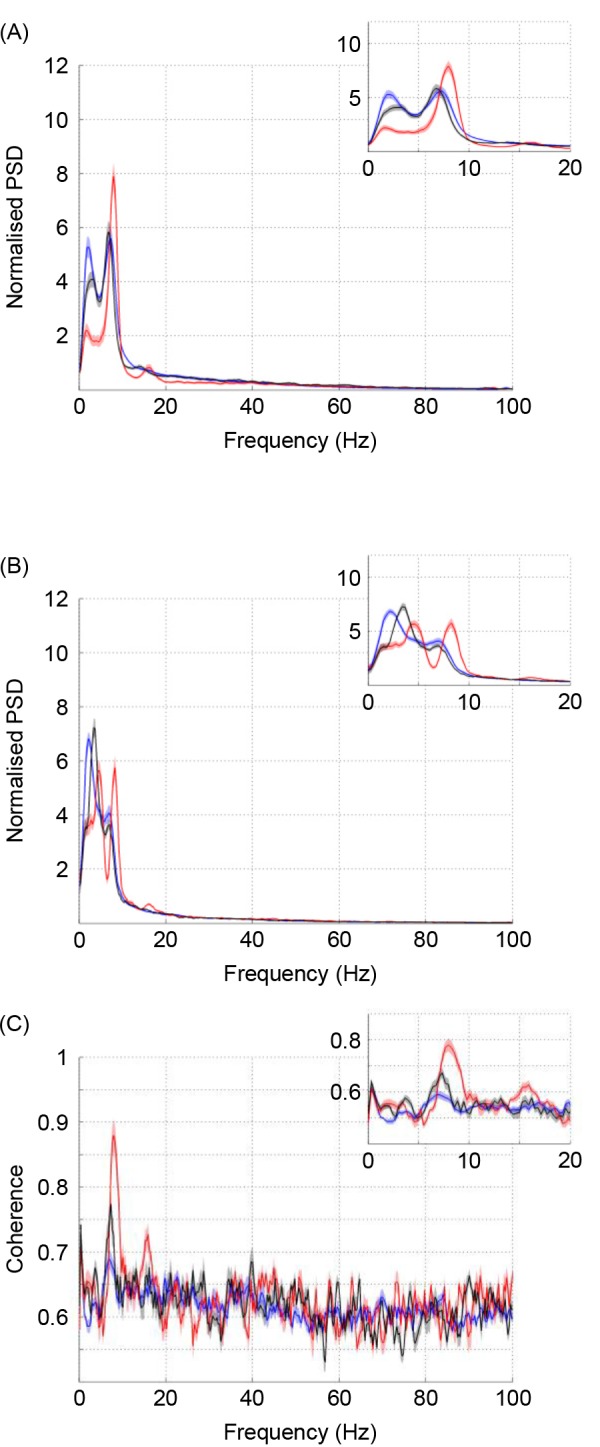
Power spectral density and coherence at different task phases. Power-spectral density (PSD) is shown for the dCA1 (A) and striatum (B), along with the coherence between these two regions (C). The traces shown are: pre-maze (blue), water-maze (red) and platform (black), ± SEM. A magnified signal (0–20 Hz) is shown in the inset. Notably, theta-frequency PSD and coherence are both elevated inside the water-maze environment. Coherence is also elevated for rats on the maze platform, albeit to lesser extent.

Normalised power spectral density (PSD) is shown for both the dCA1 and striatal brain regions (Fig 6A and 6B), alongside the coherence between these structures (Fig 6C); for each of the different task phases (pre-maze, maze, platform). A 1-way ANOVA was used to assess differences in theta-frequency PSD/coherence at each of the task phases. Theta-frequency PSD was found to be significantly elevated in the water maze, relative to the pre-maze recording (F(2,21) = 4.18, p = 0.0279), whereas striatal theta was elevated in the maze relative to both the pre-maze and platform recordings (F(2,21) = 6.88, p = 0.005). Similarly, theta-frequency coherence was significantly increased in both the maze and platform locations, relative to the pre-maze recording (F(2,18) = 12.26, p = 0.0004).

**Fig 7:**
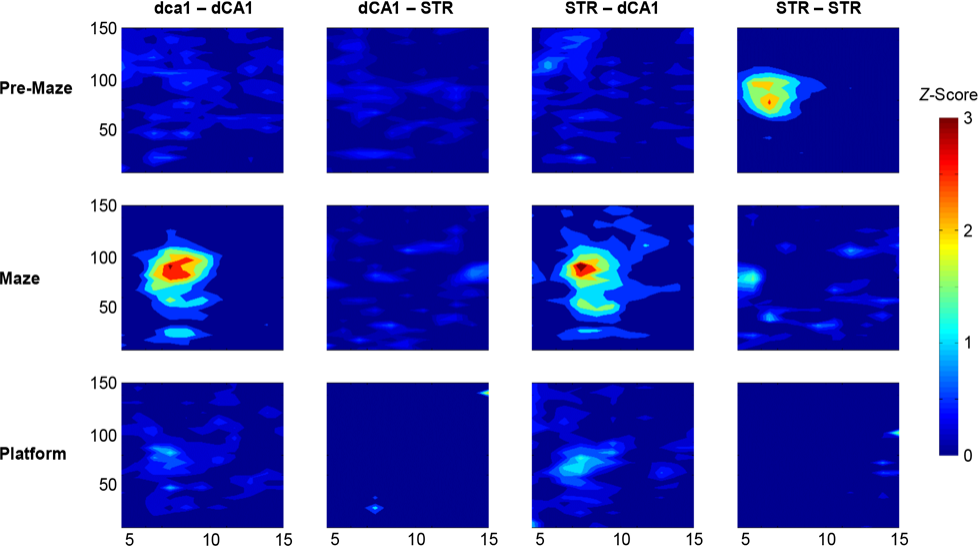
Cross-frequency modulation at different task phases. Montage depicting the theta-gamma cross-frequency modulation between and within the dCA1 and striatal (STR) brain regions, for each of the three task phases (pre-maze, maze and platform). The y-axis represents the modulated gamma frequency, whereas the x-axis is the modulating theta frequency. MI values are represented as a *z*-score following statistical comparisons with shuffled data. Notably, cross-frequency modulation is at its strongest for dCA1 gamma, for rats inside the maze. Intra-striatal cross-frequency modulation is also at its strongest when the rat is not engaged in the water-maze task (Pre-Maze; STR-STR).

In a similar manner to the theta-frequency PSD/coherence increases, the theta-gamma cross-frequency modulation was markedly increased both within and between dCA1, for recordings inside the water-maze environment (Fig 7). Although both dCA1 and striatal theta was seen to modulate dCA1 gamma, this was not observed for striatal gamma and dCA1/striatal theta. Notably, intra-striatal cross-frequency modulation was at its maximum during the pre-task baseline recordings, and had diminished throughout the execution of the task.

### Extent of Artefacts

**Fig 8:**
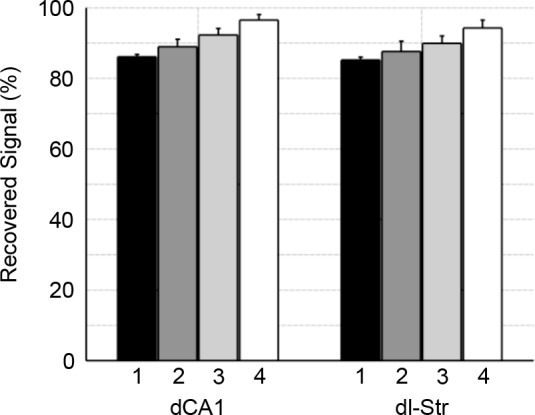
Recovered LFP following artefact removal. The mean percentage of recovered LFP + SEM is shown for both dCA1 and striatal (dl-Str) brain regions, for behavioural sessions 1–4. The percentage of recovered signal increased for both brain regions, from ~85% in session 1, to ~95% in session 4.

The percentage of the usable EEG following artefact removal was compared between each of the recording sessions, for both brain regions (dCA1 and striatum). In both brain regions the amount of recovered signal was seen to increase steadily from ~85% to 95% across the 4 training sessions (Fig 8). A significant effect of recording session was found for both dCA1 (F(3,31) = 7.59, p = 0.0007) and striatal brain regions (F(3,31) = 3.11, p = 0.0422), with multiple comparisons analysis reporting significant differences between the first and fourth session, for both brain regions.

## Discussion

This study has demonstrated a successful method for carrying out wireless recording inside a water maze. Rats were able to swim freely about the maze, and EEG was recorded and transmitted during all phases of the task. Owing to the wireless nature of the system used, EEG recording could be carried out continuously during the different task phases (pre-maze, maze, platform), without the need for manipulating the device or attaching/removing connecting cables. In addition to the increased simplicity regarding the recording set-up, the wireless system allowed recordings to take place in the enclosed sections of the maze (at the end of each of the 6 maze arms); which would not have been possible with a typical tethered recording setup. As such this method presents a practical and cost-effective solution for expanding the range of measurements currently possible in this diverse area of behavioural neuroscience.

One of the main benefits of this device over implantable systems, is its transmission range. By using a head-mount configuration, the absorption of the RF signal by water is kept to a minimum, thus allowing transmission ranges of up to 4 m using the commercial wireless system (W32; Multichannel Systems). Implantable systems are not suited for such tasks, given their tendency for a small transmission range. In order to maintain a low power consumption, these devices may be used in tandem with inductive coils [9] or Faraday cage enclosures [8]. Furthermore, their RF signal is further attenuated by the system enclosure, overlying tissues, and to a certain degree, its immersion inside water. To elaborate this fact, the only implantable device that has demonstrated to successfully record in water, allowed 1 –channel to be transmitted at a range of 30 cm [10]. The benefit of the current method is that it is the first one to facilitate both multichannel wireless recording at a range that is suitable for typical rat water-maze tasks (e.g. Morris water maze, double-H maze, etc.).

The waterproof attachment was designed to solve 2 problems inherent with water-maze recording. The first was to prevent water from reaching the EEG connector, battery and the wireless system itself. This was achieved by a combination of the latex finger-cot, Vaseline, and by the shape of the attachment and headstage socket. In order to reduce the time taken to attach the system, Vaseline was seen as a fast, cheap and reliable means of providing a rapid sealant. After connecting the system to the head of rats, it took only seconds to apply Vaseline around the base of the implant. As such there were no leakages observed at any part of the behavioural task, in any of the rats. Despite a success rate of 100% (8 rats × 22 trials each), it cannot be ruled out that with further testing and/or alternate paradigms leakages and/or breakages may occur. However the current success rate is encouraging to warrant further reliable recording using this method.

The second problem that needed to be addressed was the movement artefacts from the rat shaking inside the water maze. Movement artefacts are typically present in both wired and wireless systems during recordings in rats, which results from the implant-recording connector being disturbed (e.g. from grooming, the rat shaking, hitting the system/cable on the side of the arena, etc.). The rat shaking behaviour as observed inside the water maze were severe enough to damage previous (unreported) iterations of this device; i.e. Omnetics connectors (including their plastic housing and metal pins) were torn apart. Although this has not been documented in previous studies that featured water-maze EEG recording [1–5,10], it was considered severe enough in this study to require special attention regarding the design of the connector. As such, the connector was designed to redistribute these forces away from the EEG connector whilst protecting it from damage. This was achieved by a) protecting the connector inside a metal thimble, and b) physically screwing the thimble into the implant, allowing shaking forces to be moved away from the connector itself and onto these screws. Given the severity of the rat shaking forces, a complete removal of artefacts was not possible. Notably, the rat shaking behaviour had decreased steadily across the training sessions, resulting in a usable signal ranging from ~85% (session 1) to ~95% (session 4). This can be attributed to a) the rat spending less time inside the water maze, and b) the rat becoming habituated to the task and system. The amount of recovered signal was slightly lower in the dl-Str electrode, which was to be expected given the comparatively higher impedance of this electrode.

The waterproof connector was also designed to minimise the amount of handling required when attaching the system to the rat; which is important for tasks of this kind which inherently result in acute stress for the animal. As such the latex finger-cot and elastic band was applied prior to attaching the system/connector to the head of the rat. All that was required was to connect the EEG plug, screw in the thimble, roll down the finger-cot and apply Vaseline. With relatively little practice this could be achieved in typically less than a minute. When comparing the performance between the recording and non-recording groups of rats, no significant differences were observed across a number of measures, including the time taken to reach the goal and the number of errors made.

Many of the LFP findings in this study had reflected similar observations in rats during exploratory behaviour, such as enhanced theta-frequency PSD during movement and spatial exploration [11,12]. An enhanced theta-frequency coherence has been observed during working memory between the hippocampus and various structures, including the prefrontal cortex during working memory [13,14], the striatum during place memory, [21], and the entorhinal cortex during declarative memory [22]. Theta-gamma cross-frequency modulation [23] is being increasingly probed in dCA1, and has been proposed as a mechanism for working memory [24]. More recently, a dynamic modulation of this process both within and between dCA1 and the striatum has been demonstrated following discrete task phases inside a T-maze [16]. The observations of increased theta-gamma modulation inside the water-maze support these prior observations, in that it may be a feature of goal-orientated exploratory behaviour. Furthermore, it highlights more prominently a modulation of dCA1 gamma activity as opposed to striatal gamma activity. Functionally, the cross-structure modulation may be due in part to the underlying hippocampal theta rhythm, which was shown to reach a maximum coherence with striatal theta during the swimming task.

Although single-unit recording was not demonstrated in the current study, it is theoretically possible with the current equipment, given that the wireless system used sampled at 10 KHz, and is capable of the detection of spikes and action potentials. Thus alternate electrodes fit for the detection of such waveforms may allow for the detection of spikes inside the water maze.

Taken together this waterproofing technique can be utilised to expand the range of measurements capable in water-maze environments, which may help to pave the way for a greater functional understanding of brain regions during goal-orientated behaviour.

## Supporting Information

S1 Video Simultaneous EEG recording and rat swimming during early training. (MP4)

S2 Video Simultaneous EEG recording and rat swimming during middle of training. (MP4)

S3 Video Simultaneous EEG recording and rat swimming during late training. (MP4)
